# Sex-related differences in endothelium-dependent vasodilation of human gingiva

**DOI:** 10.1186/s12903-022-02186-2

**Published:** 2022-05-13

**Authors:** János Vág, Tamás László Nagy, Barbara Mikecs

**Affiliations:** Department of Conservative Dentistry, Faculty of Dentistry, Semmelweis University, Szentkirályi utca 47, Budapest, 1088 Hungary

**Keywords:** Sex, Gingiva, Microcirculation, Nitric oxide, Acetylcholine, Endothelium

## Abstract

**Background:**

Sex hormones influence circulation, periodontitis, and wound healing. The aim of the study was to compare the endothelium-dependent and independent vasodilation in human gingiva in men and women.

**Methods:**

Gingival blood flow was evaluated in twelve male and twelve female subjects with healthy gingiva and no systemic conditions after acetylcholine or nitric oxide donor (NitroPOHL). Agonists were administered into the gingival sulcus at the right secondary incisor (test site). Regional gingival blood flow (GBF) was imaged by Laser Speckle Contrast Imager from the marginal gingiva to the mucogingival junction in four consecutive regions (coronal, midway1, midway2 and apical). Blood flow was expressed in Laser Speckle Perfusion Unit (LSPU). The absolute maximal blood flow change (Dmax), the area under the blood flow curve (AUC), and the time to peak (TTP) were calculated.

**Results:**

Males had higher baseline GBF than females (257 ± 18.2 vs. 225 ± 18.8 LSPU, *p* < 0.001). Acetylcholine and NitroPOHL significantly increased the GBF in all test regions. The Dmax after the acetylcholine was reduced apically compared to the coronal (90 ± 13 LSPU vs. 117 ± 7 LSPU, *p* < 0.01), but it was similar after NitroPOHL (78 ± 9 LSPU vs. 86 ± 6 LSPU, *p* = 0.398) in both sexes. The Dmax and AUC were higher, and the TTP was smaller in men in most regions after acetylcholine but not after NitroPOHL.

**Conclusion:**

In the human gingiva, the endothelium-independent vasodilation propagates without attenuation in the line of the vascular supply in both sexes. At the same time, the endothelium-dependent ascending vasodilation attenuates similarly in men and women. However, men had more pronounced endothelium-dependent vasodilation than women. Therefore, it might contribute to the increased severity of periodontal disease in men.

***Trial registration*:**

The study was registered with ClinicalTrials.gov on 09.06.2021 (NCT04918563).

## Background

The increased metabolic activity or temporary ischemia in tissue induces vasoactive factors release and results in local vasodilation (i.e., functional and reactive hyperemia). The vasodilation should ascend (i.e., spread) to the proximal feed artery and arterioles to ensure the blood supply of the distal microcirculatory network [1]. Recently, spreading vasodilation and vasoconstriction evoked by nitric oxide or epinephrine was observed in human gingiva [2, 3], which could have an impact on flap survival and the spatial progression of periodontitis. However, some evidence suggests that microcirculation of the human gingiva might be affected by sex. During the first week of healing and after applying modified coronally advanced tunnel technique for gingival recession coverage, gingival blood flow was significantly higher in males than in females [4]. In addition, hyperemia was higher in men after brief occlusion of gingival vessels [5]. There is also evidence that palatal mucosa wound healing time varies depending on the testosterone and progesterone level, menopause, and hormonal replacement therapy [6]. Similar to the gingiva, after ligation of the hindlimb in mice, females showed impaired flow recovery due to decreased collateral remodeling, less angiogenesis, impaired vasodilator response, and increased vasoconstrictor activity compared to males [7].

Men have a higher prevalence and severity of periodontal disease than women [8]. Evidence suggests that this difference might relate to a difference in endothelial function. A common way to assess endothelial dysfunction is to measure the integrity of the flow-mediated vasodilation (FMD) by the post-occlusive reactive hyperemia (PORH) test [9]. Namely, after the release of occlusion of the vessels, the blood velocity rapidly increases. The increased speed raises the shear stress on the endothelial walls, resulting in the release of vasodilators [10, 11]. In patients with advanced periodontitis, the FMD at the brachial artery is reduced [12]. Sex differences in PORH were observed in several studies in large arteries [13, 14] and the microcirculation [15, 16], including the gingiva [5]. However, several mechanisms could mediate the PORH (e.g., nitric oxide, prostanoids, endothelium-derived hyperpolarizing factor) in various tissues [10, 11, 17, 18]. Instead of the PORH test, a specific agonist more explicitly distinguishes between endothelium-dependent and independent vasodilation. Acetylcholine can assess the integrity of the endothelium-dependent vasodilation because it releases nitric oxide from the endothelium [19, 20]. The released nitric oxide then relaxes the underlying vascular smooth muscle cell [20]. Contrarily, nitrovasodilators such as sodium nitroprusside and nitroglycerin release nitric oxide by biotransformation in tissues, directly relaxing the vascular smooth muscle cell [21]. Therefore, nitric oxide donors can be used to evaluate endothelium-independent vasodilation [19].

The primary aim was to characterize the endothelium-dependent and independent spreading vasodilation in terms of the attenuation of remote response in human gingiva. The secondary purpose was to compare the spreading vasodilation between men and women. The null hypothesis was that neither acetylcholine (endothelium-dependent) nor nitric oxide donor (endothelium-independent) induced spreading vasodilation are not significantly different between women and men.

## Methods

Twelve female and twelve male volunteers were involved in the study. The age was between 20 and 32 years (mean of 24.8 years) for women and between 20 and 30 years (mean of 23.8 years) for men. Only young subjects were selected to avoid the possible effect of age and hormonal changes on the gingival blood flow (GBF). The study was carried out following the Declaration of Helsinki. Ethical approval was granted by the Hungarian National Public Health and Medical Officer Service (20104/2017/EÜIG). The study was registered with ClinicalTrials.gov at 09.06.2021 (NCT04918563). Each subject received written information about any possible risk and details of the measurement. Signed informed consent was obtained. Volunteers were selected based on the criteria listed in Table 1. In addition, the participants were not allowed to eat, drink and brush their teeth 60 min before the measurement to avoid any extrinsic and intrinsic stimulus affecting the GBF.

**Table 1:** Inclusion and exclusion criteria

Inclusion criteria	Exclusion criteria
Good systemic health	Systemic medication
Good oral health	Contraceptives
	Pregnancy
	Smoking
	Gingivitis
	Caries
	Fillings and prosthetics restoration at the upper front teeth

The sample size was estimated determined by previous studies [2, 4, 5]. The GBF changed from 180 LSPU (standard deviation: 52 LSPU) to 251 LSPU (standard deviation: 67 LSPU) after NitroPOHL [2]. In addition, the mucogingival flap blood flow after surgery in the hyperemic period was 85% higher in males than females [4]. GBF after the PORH test was 18% higher in males than in females [5]. An appropriate sample size analysis indicated that 10 participants were needed to detect significant differences (approximately 30% between sex groups with an effect size of 1.02) in GBF at *p* < 0.05 and 80% power.

**Fig. 1:**
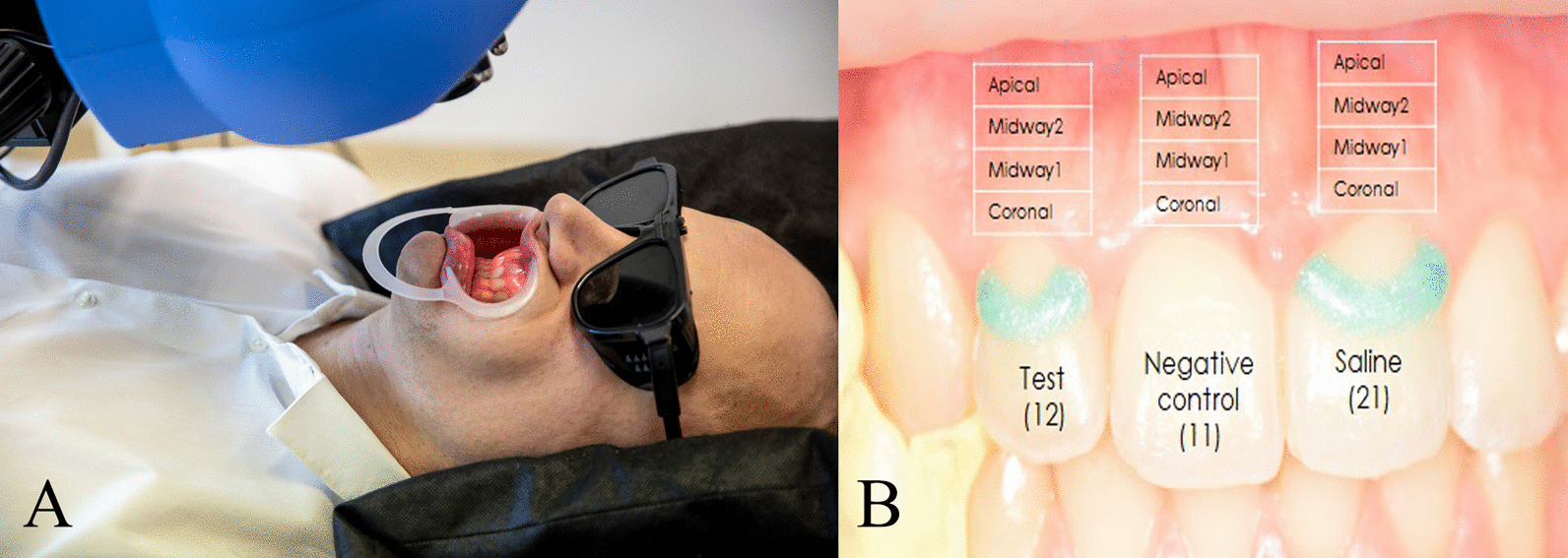
Measurement setup. The patient's head is fixed with a vacuum pillow, and the lips are retracted with a cheek retractor (**A**). Silicon bite for stabilizing the jaws (left side), semi-circular wells on the labial surface of the upper right second incisor, and upper left first incisor. Coronal, Midway1, Midway2, Apical regions of interest (**B**)

The patients were seated in a standard supine position in a dental chair. They were allowed to accommodate for 15 min in a quiet room with a temperature between 24 and 26 °C. Systolic and diastolic blood pressure and heart rate were measured with an automatic blood pressure monitor (Omron M2, Omron Healthcare Inc., Kyoto, Japan) on the left arm three times during each session, after patient arrival, and immediately before and after the GBF measurement. The patient's head was fixed by a vacuum pillow (Spandex®, Hager&Werken, Germany) (Fig. 1A). The lips were retracted carefully with a lip retractor (OptraGate, Ivoclar Vivadent AG, Liechtenstein), and the jaw was stabilized using a silicone bite.

The GBF was measured by Laser Speckle Contrast Imager (LSCI, PeriCam PSI HR System, Perimed AB, Stockholm, Sweden) and expressed in the arbitrary unit as laser speckle perfusion unit. The LSCI illuminates the object with infrared red laser light (785 nm). The laser light reflects from static objects and forms a speckle pattern. However, moving cells blur this pattern proportionally to their velocity. By calculating the contrast of the captured image, LSCI can assess the microcirculation in a non-contact and non-invasive manner with high spatial and temporal resolution. The adaptation of this method to the human gingiva revealed high accuracy [22]. The measurement area was set to 2 × 3 cm and the resolution to 0.05 mm/pixel.

Two semi-circular wells were fabricated on the surface of the teeth with light-cured resin-based barrier material (Optra Dam green, Ultradent Products Inc., USA) (Fig. 1B). A well was made at the labial surface of the upper right second incisor (test site). Another well was placed at the upper left first incisor (saline site). No well was formed at the upper right first incisor, serving as a negative control. At the first measurement, 3 µl acetylcholine in 10 mg/ml (acetylcholine chloride A2661-25G, Sigma-Aldrich Chemie GmbH, Switzerland) was applied on the test site. On a second visit, 3 µl nitric oxide donor—NitroPOHL in 1 mg/ml (NitroPOHL®, Pohl-Boskamp GmbH, Germany) was administered. Agonist concentrations were chosen based on the previous findings. Acetylcholine was used safely in 1, 10, 20 mg/ml concentrations in human studies on the skin locally in healthy patients and patients with cardiovascular disease [23–27]. Acetylcholine chloride was diluted in sterile physiological saline in an Eppendorf vial. In our previous experiment on the human gingiva, the 1 mg/ml NitroPOHL induced significant hyperemia without any systemic effect in the keratinized gingiva [2]. NitroPOHL® was delivered directly from the sterile ampoule.

A random number generator decided the order of the applied solutions. One of the co-authors (B.M.) generated the allocation sequence. Then, B.M. enrolled the participants and assigned them to interventions. According to the crossover design, the subjects were randomly assigned to one of the following sequences. In group 1 (six men, six women), the acetylcholine was applied first, and in group 2 (six men, six women), the NitroPOHL was applied first. The volunteers were unaware of the applied solutions. On the saline site, 3 µl of physiological saline was added. Before their application, both solutions and the application syringes were preheated to 37 °C in a block heater (Dry Block Thermostat DBI-100, Boeckel GmbH, Hamburg, Germany). Minimally 1 week was between the two measurement sessions. Both solutions were applied with the same method. No changes were applied in the protocol after the allocation of the subjects and the commencement of the trial.

After the patient arrived for the measurement session, the blood pressure and pulse were recorded (patient arrival). Then, the wells were placed on the teeth, and the LSCI head was adjusted. Then the subjects rested in the dental chair for 15 min, defined as resting period. After that, the blood pressure and pulse rate were measured again (before LSCI). Next, GBF recording started and was recorded for 1 min (the baseline GBF) followed by applying solutions into the corresponding wells with a Hamilton syringe (Model 75 RN SYR, Hamilton, Switzerland). After that, GBF changes were recorded for a further 15 min. After GBF recording, the third blood pressure and pulse rate measurements were done (after LSCI). After GBF recording, the healthy condition of the gingiva was reaffirmed by measurement of the gingival crevicular fluid (GCF) and the pocket depth. The GCF was collected with PerioPaper at each investigated tooth two times for 5 s. The wet of the paper strip was evaluated with Periotron 8000 (PeriCam PSI HR, Perimed AB) and expressed in Periotron Unit (PU). The probing depth and the width of the keratinized gingiva were measured with a periodontal probe (UNC 15, Hu-Friedy, Chicago, IL, US). The same investigator for all subjects carried out the experimental procedure.

The analysis of the LSCI recording was done by another researcher. Four regions of interest (ROI) were defined above each investigated tooth (Fig. 1B) on the keratinized gingiva by the ROI tools of the Pimsoft Software (PeriCam PSI HR, Perimed AB). Each was 1 mm high, the coronal, the midway1, the midway2, and the apical.

The primary outcome was to measure the absolute blood flow change from the baseline by the time and by region after applying the test solutions. The secondary outcome was to compare these changes between agonists and sexes. No changes were made in outcome measures during or after the trial commenced. The study was planned for 1 year. All 48 measurements were successfully done; therefore, it is ended as planned. No patient was excluded from the study during the study period. The planned number of the patients was assigned to the intervention. No harm or any unintended effect appeared in either group.

### Statistics

The systolic and diastolic blood pressure, heart rate, and GBF in the text and graphs are presented in mean ± SE. The GCF is shown as median with the first and the third quartile (Q1 and Q3). The time-related change in GBF was calculated as the difference between the actual value and its corresponding baseline value. The GBF at the test site was also controlled by the change in GBF at the saline site by calculating their difference. The maximal blood flow change (Dmax) value was calculated by taking the maximum GBF change for each case during the 15-min agonist application period. The Time to Peak (TTP) was the exact time of the maximum response. The total hyperemic response was calculated by taking the area under the curve (AUC) of GBF changes. The blood pressure (systolic, diastolic), pulse rate, GBF were statistically analyzed by Linear Mixed Model. The effects of the agonist, the sex, the region, the time, and the interaction were included in the model as fixed effects. The region, agonist, and time were also included as random factors. A moderate correlation was found between baseline GBF and systolic blood pressure (before LSCI). Therefore, the systolic blood pressure was included as a covariate in comparing the baseline GBF. No correlation was found between baseline and Dmax. Therefore, the absolute change was compared between sex groups instead of percent change [28], and the baseline covariate was included in the model [29]. Bonferroni adjustment was applied in pairwise comparison, considering *p* < 0.05 as a significant difference. GCF was compared by Generalized Linear Mixed Model. Pearson correlation coefficient was calculated to assess the relationship between parameters. All statistics were made in IBM SPSS Statistics, Version 27 (Armonk, NY: IBM Corp., USA).

## Results

### Baseline parameters and change at the saline site

**Fig. 2:**
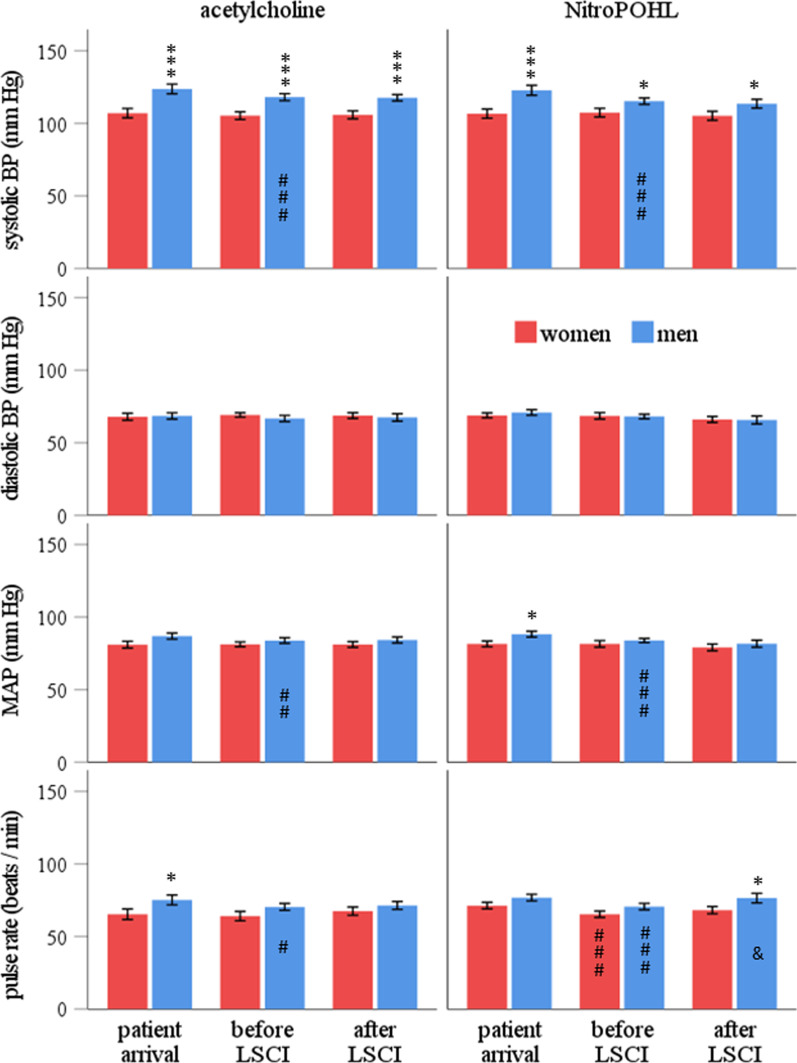
Systolic, diastolic, mean blood pressure and pulse rate were recorded upon arrival of the patient, before LSCI, and after LSCI measurement. Each woman (red column) and man (blue column) visited the clinic twice (sessions), once for the acetylcholine and once for the NitroPOHL application. BP indicates blood pressure; MAP stands for mean arterial blood pressure. *above the columns indicates significant differences between women and men, *p < 0.05, ***p < 0.001. # in the columns indicates significant differences between the patient arrival and the before LSCI measurement (i.e., change during the resting period), # *p* < 0.05, ## *p* < 0.01, ### *p* < 0.001. & indicates significant differences in the blood flow measurement between the before and after LSCI measurement (i.e., change during the LSCI measurement), & *p* < 0.05

Men had significantly higher (Fig. 2) systolic blood pressure than women in both acetylcholine and NitroPOHL sessions and all three measurement periods. The diastolic blood pressure was not different between sexes and sessions. The MAP was significantly higher in men only at patient arrival. The pulse rate was higher in men at patient arrival in acetylcholine session and after LSCI in NitroPOHL session (Fig. 2.).

**Fig. 3:**
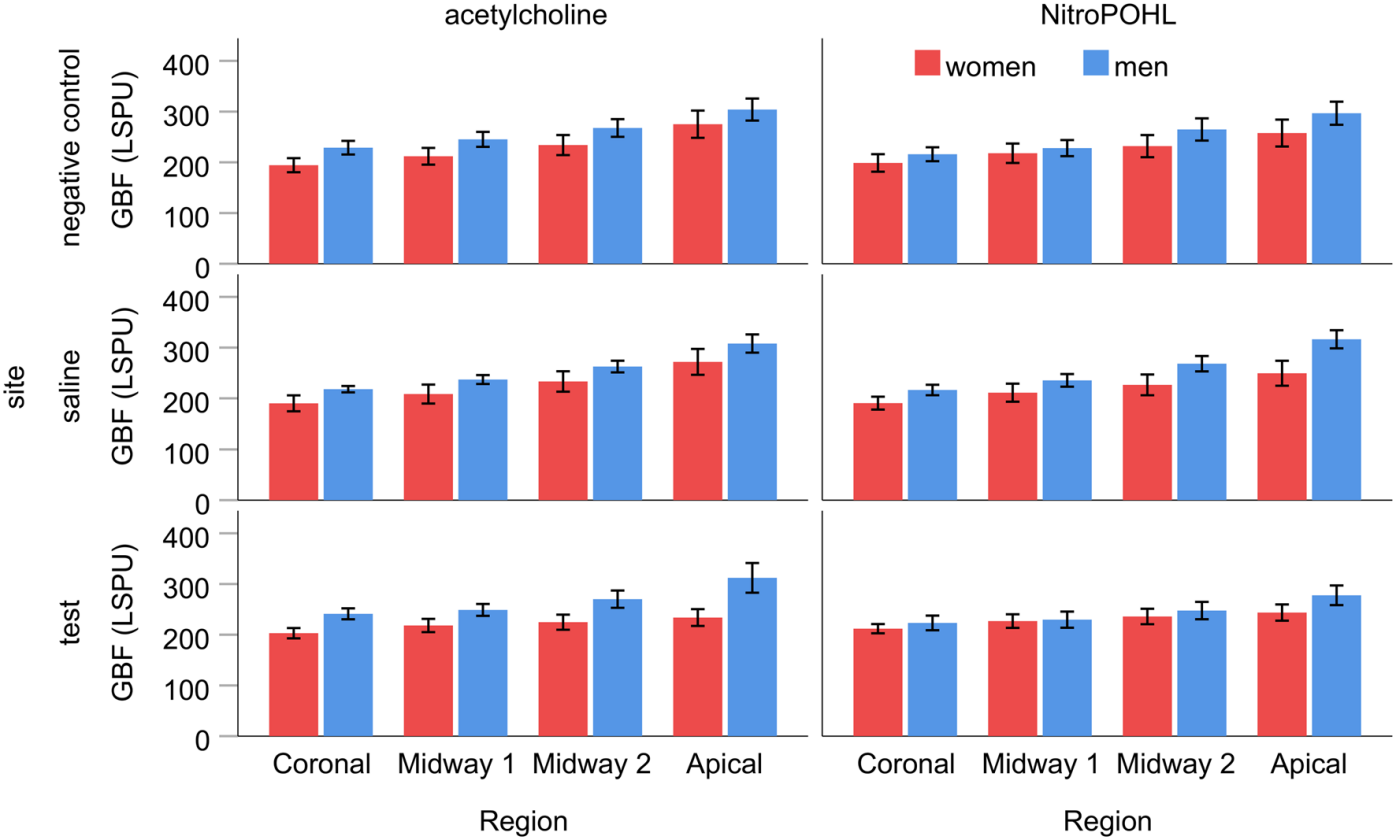
The comparison of baseline gingival blood flow (GBF) between sex groups at negative control, saline, and test site in different regions. GBF is expressed in the laser speckle perfusion unit (LSPU). The baseline GBF was not significantly different between agonists and sites. However, the GBF was gradually increased from coronal to apical. The men had higher baseline GBF than women in all sites and regions

The GCF was not different significantly between the sexes (*p* = 0.995), agonists (i.e., sessions, *p* = 0.728) and sites (*p* = 0.086, Table 2.). The baseline GBF (Fig. 3.) was not significantly different between agonists (i.e., sessions) (*p* = 0.126) and between sites (*p* = 0.968) after controlling systolic blood pressure (covariant). However, the region effect was significant (*p* < 0.001), indicating that GBF was gradually increased from coronal to apical by 15.5 ± 6.7 LSPU (*p* < 0.05), 20.7 ± 8.1 LSPU (*p* < 0.05), and 31.6 ± 9.9 LSPU (*p* < 0.01). And site x agonist x sex interaction (*p* < 0.01) was significant. Additionally, men had higher baseline GBF than women (257 ± 18.2 vs. 225 ± 18.8 LSPU, *p* < 0.001) but the difference varied between sites and agonists (i.e., session) from 8.6 ± 10.3 LSPU to 37.8 ± 12.1 LSPU.

**Table 2:** Comparison of baseline gingival crevicular fluid (in Periotron Unit)

Site	Session by agonist			
	ACH		NO	
	women	Men	women	men
*Negative*				
Valid N	12	12	12	12
Median	3.8	4.0	2.5	2.3
Q1	2.8	1.8	2.3	1.8
Q3	6.5	9.8	3.8	8.5
*Saline*				
Valid N	12	12	12	12
Median	3.0	2.5	4.0	3.5
Q1	2.3	1.0	1.5	2.5
Q3	5.0	7.0	5.3	5.0
*Test*				
Valid N	12	12	12	12
Median	4.8	3.3	4.5	4.3
Q1	3.0	2.5	2.8	2.5
Q3	6.0	10.3	10.0	6.8

**Fig. 4:**
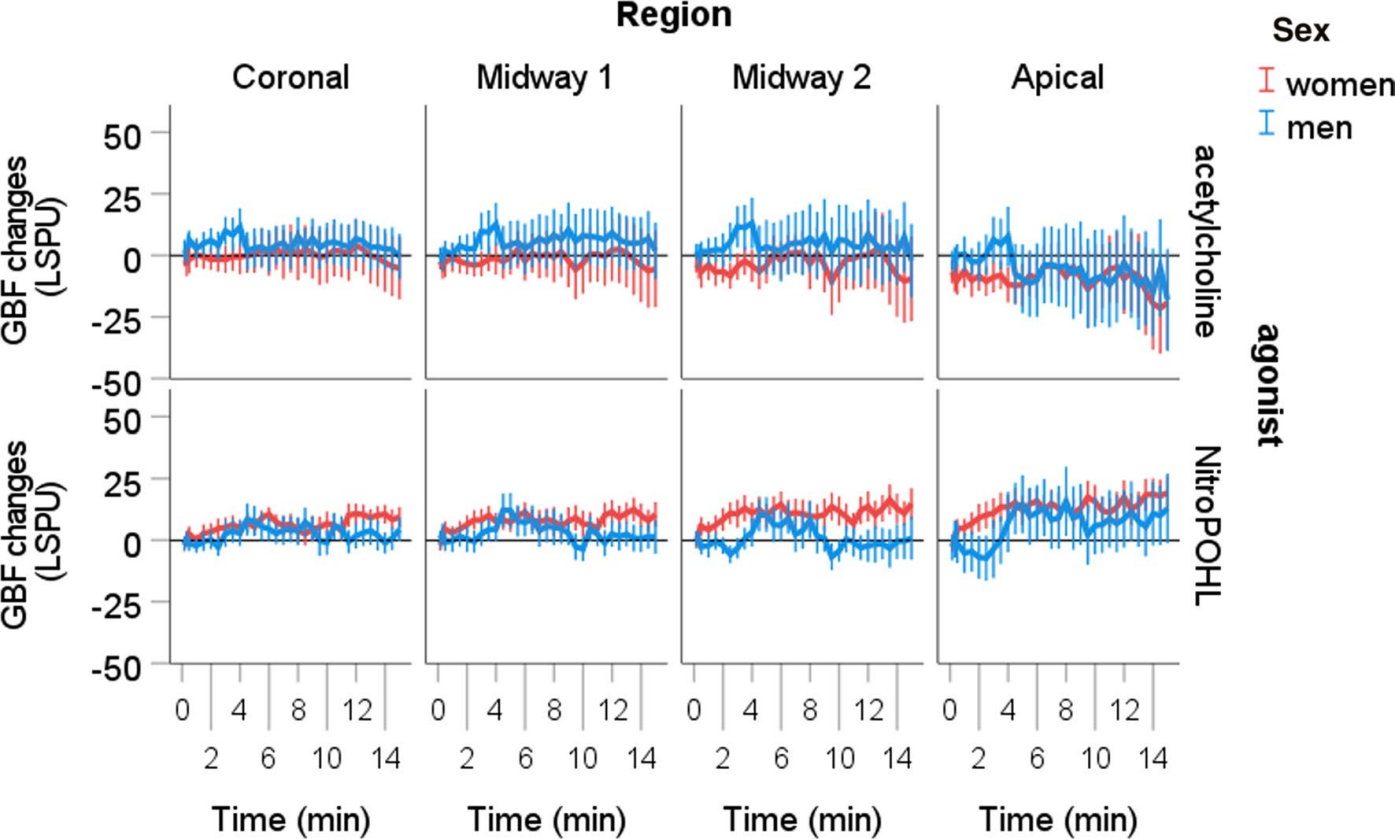
The changes of gingival blood flow (GBF) at the negative control site in different regions over time. Negative control means no intervention was applied here. The change from the baseline was normalized to the saline site. Pairwise comparison between women and men revealed no significant difference at any time points

In the negative control site (no intervention was applied), there was a significant interaction of the time x agonist x sex x region (*p* < 0.01) in GBF. The GBF fluctuation by the time was within a range of -20 and 20 LSPU, which was less than 10% of the baseline value (Fig. 4).

### Change in systemic circulatory parameters during the measurement session

In men, the systolic blood pressure, MAP, and pulse rate significantly decreased during the resting period (patient arrival vs. before LSCI) in both sessions (Fig. 2.). In women, the pulse rate decreased significantly in the NitroPOHL session during the rest (Fig. 2.). A significant change (5.8 ± 2.3 1/min) was found only in men's pulse rate during the NitroPOHL application (before vs. after LSCI).

### GBF change after agonist administration

**Fig. 5:**
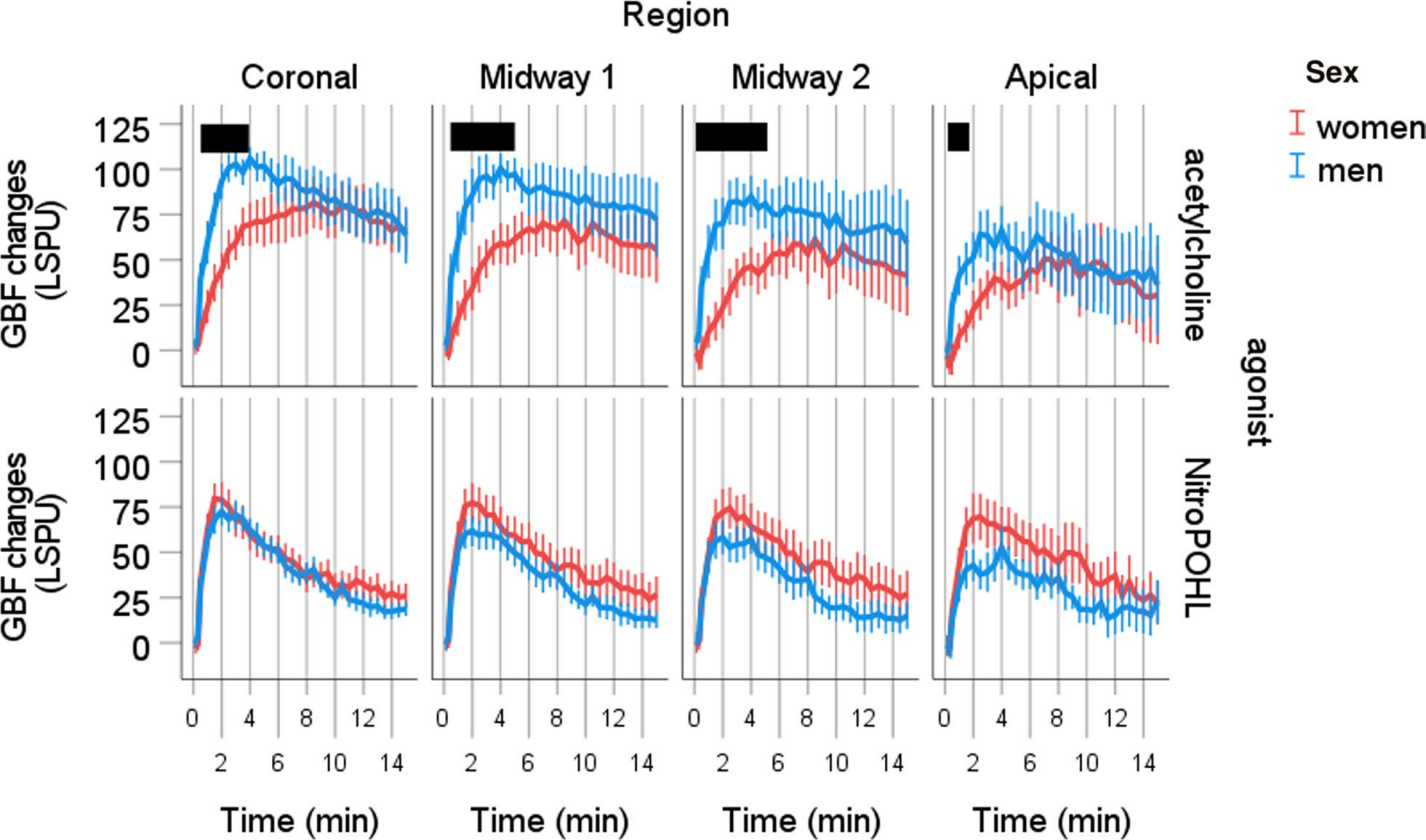
Blood flow changes from the baseline normalized to the saline site at different regions after acetylcholine or NitroPOHL administration in different regions. The black boxes indicate the duration of significant differences between sexes at least *p* < 0.05

In all regions, the time x sex x agonist interaction was significant (*p* < 0.001). The GBF changes by the time in the four regions are shown in Fig. 5. In the coronal region, men had significantly higher GBF after acetylcholine at 30 (*p* < 0.05), 60 (*p* < 0.05), 90 (*p* < 0.01), 120 (*p* < 0.01), 150 (*p* < 0.01), 180 (*p* < 0.01), 210 (*p* < 0.05) and 240 s (*p* < 0.05) (Fig. 5). In the midway1 region, men had significantly higher GBF after acetylcholine at 30 (*p* < 0.05), 60 (*p* < 0.05), 90 (*p* < 0.05), 120 (*p* < 0.01), 150 (*p* < 0.05), 180 (*p* < 0.05), 210 (*p* < 0.05), 240 (*p* < 0.01), 270 (*p* < 0.05) and 300 s (*p* < 0.05). In the midway2 region, men had significantly higher GBF after acetylcholine at 10 (*p* < 0.05), 20 (*p* < 0.05), 30 (*p* < 0.01), 60 (*p* < 0.01), 90 (*p* < 0.01), 120 (*p* < 0.01), 150 (*p* < 0.01), 180 (*p* < 0.01), 210 (*p* < 0.01), 240 (*p* < 0.01), 270 (*p* < 0.05) and 300 s (*p* < 0.05). In the apical region, men had significantly higher GBF after acetylcholine at 20 (*p* < 0.05), at 30 (*p* < 0.01), 60 (*p* < 0.01), 90 (*p* < 0.05), and 150 s (*p* < 0.01). No difference was observed between sexes after NitroPOHL in any regions.

### The Dmax, the TTP, and the AUC after agonist administration

**Fig. 6:**
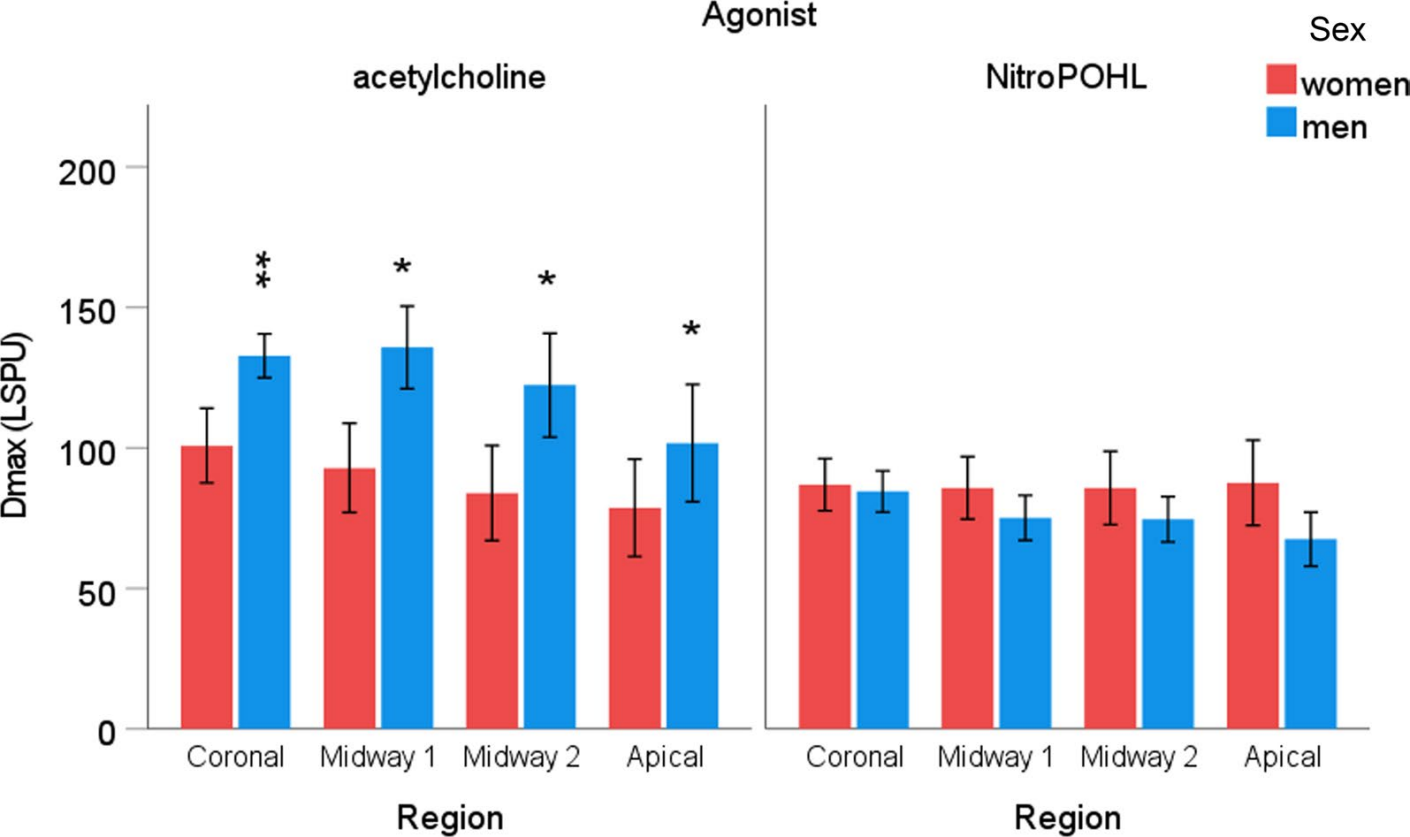
The maximal change in gingival blood flow normalized to the saline site (Dmax) in the different regions after acetylcholine or NitroPOHL in women and men. * indicates significant differences between sexes, *p < 0.05, **p < 0.01

The sex x agonist x region interaction was not significant for the **Dmax** (Fig. 6). The significant agonist x region interaction (*p* < 0.05) indicated that acetylcholine caused a gradually decreasing Dmax response; coronal, 117 ± 7 LSPU, midway1, 114 ± 10, midway2, 103 ± 12 LPSU, apical, 90 ± 13 LSPU. Contrarily, the NitroPOHL caused similar vasodilation in all regions (86 ± 6, 80 ± 7, 80 ± 7, 78 ± 9). The significant agonist x sex interaction (*p* < 0.05) revealed that acetylcholine-induced vasodilation was higher in men (123 ± 14 LSPU) than in women (89 ± 14 LSPU). However, NitroPOHL induced similar vasodilation in both sexes (75 ± 7 LPSU, 87 ± 11 LSPU).

**Fig. 7:**
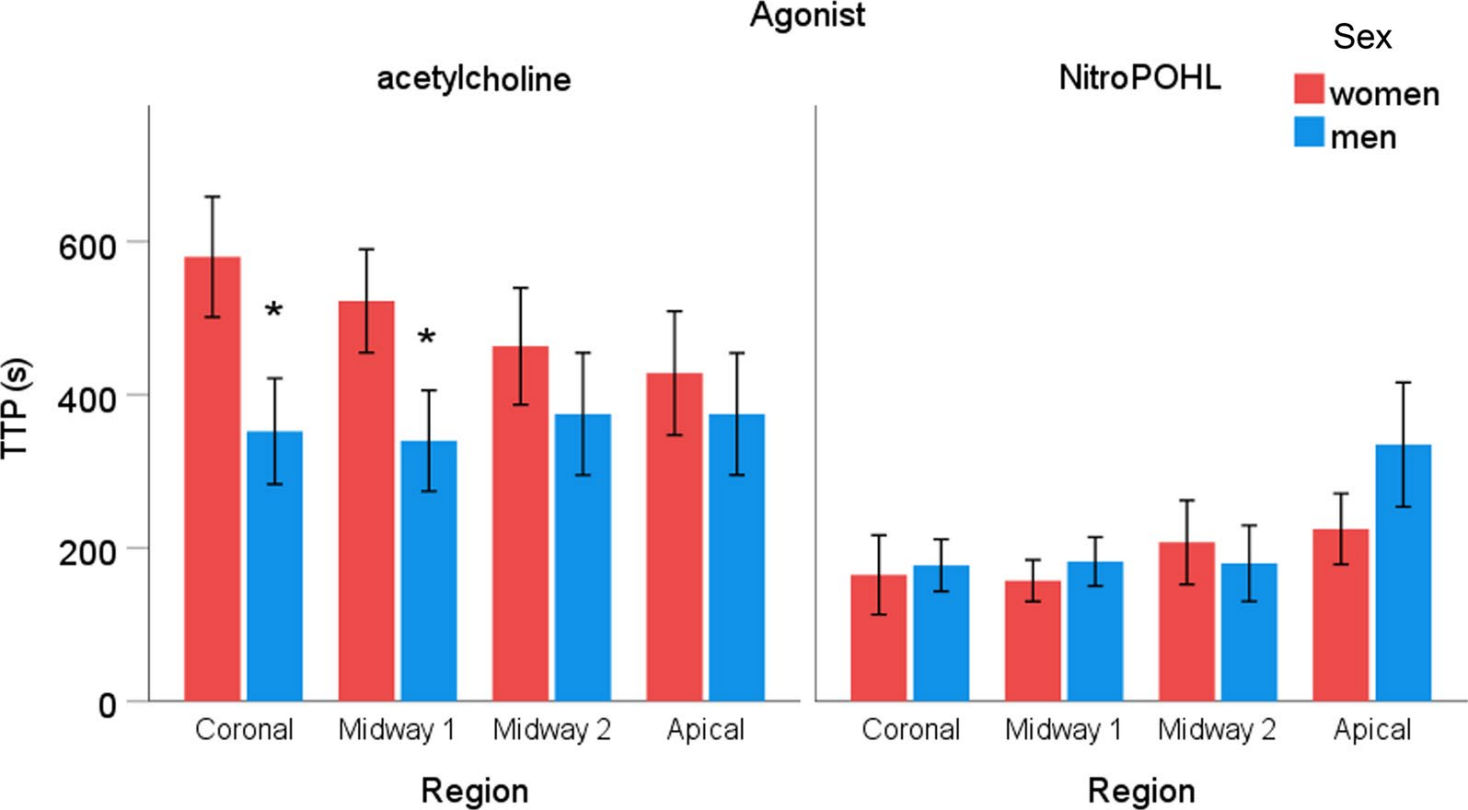
The time to reach the peak (TTP) in the different regions after acetylcholine or NitroPOHL in men and women. * indicates significant differences between sexes, *p* < 0.05

The sex x agonist x region interaction was not significant for the **TTP** (Fig. 7). The significant agonist x region interaction (*p* < 0.01) indicated that the TTP of the NitroPOHL response was smaller than that of the acetylcholine response in the coronal (148 ± 18 s vs. 394 ± 50 s, *p* < 0.001), midway1 (153 ± 17 s vs. 396 ± 44 s, *p* < 0.001), and midway2 (161 ± 21 vs. 340 ± 51 s, *p* < 0.001) regions. In contrast, the difference was diminished apically (240 ± 37 s vs. 325 ± 51 s, *p* = 0.142). Acetylcholine-induced hyperemia was faster in men than in women in the coronal and apical regions. Contrarily, no significant difference in TTP was observed between sexes after NitroPOHL.

**Fig. 8:**
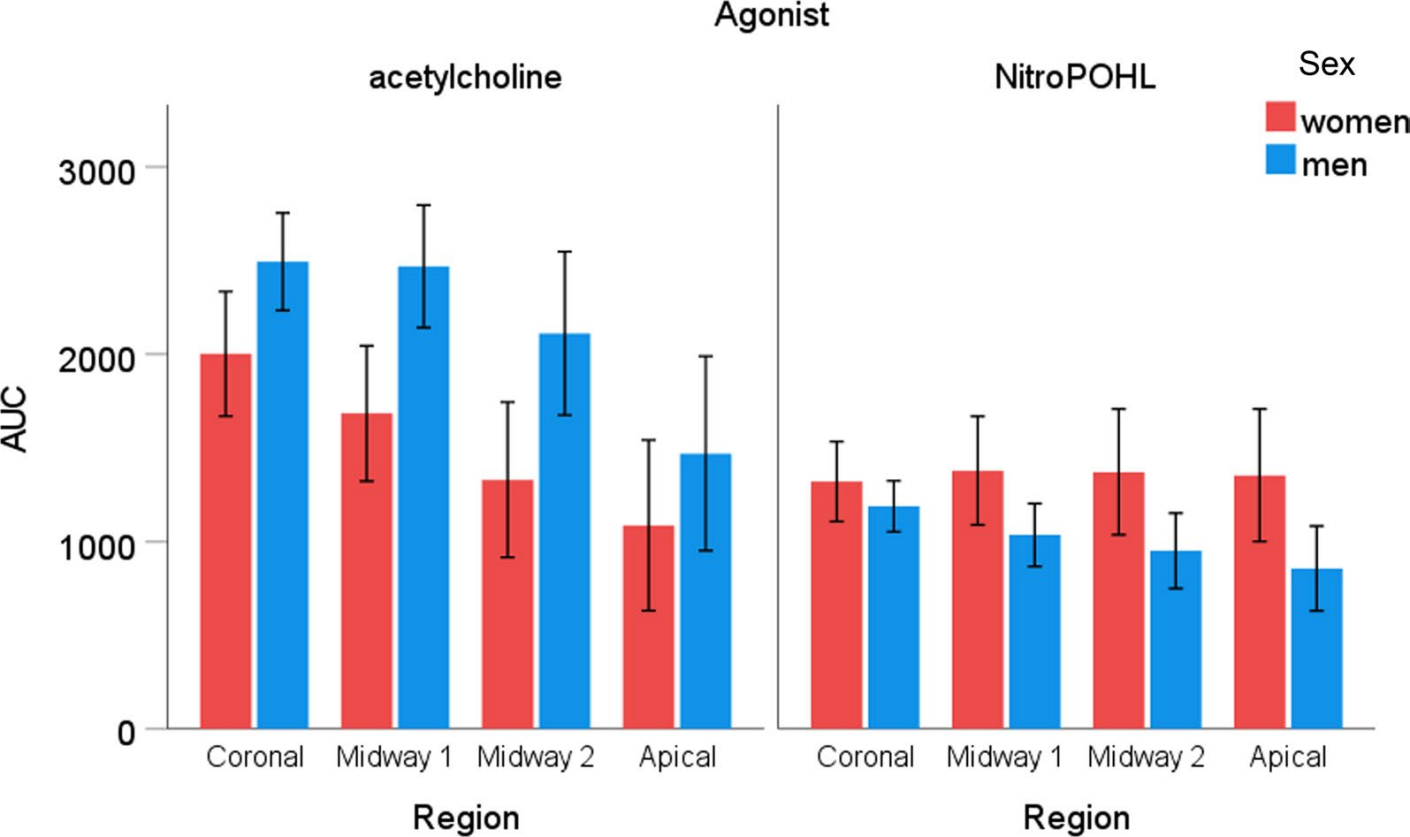
The area under the curve of blood flow changes (AUC) as a function of time normalized to the saline site in different regions after acetylcholine or NitroPOHL in men and women. * indicates significant differences between sexes, *p* < 0.05

The sex x agonist x region interaction was significant for the **AUC** (*p* < 0.05) (Fig. 8). Acetylcholine induced higher AUC than NitroPOHL in men at the coronal (2494 ± 250 vs. 1189 ± 130, *p* < 0.001), midway1 (2469 ± 313 vs. 1036 ± 161, *p* < 0.001) and midway2 (2111 ± 418 vs. 952 ± 193, *p* < 0.05) regions. The two agonists produced similar hyperemia in the apical region in men (1417 ± 496 vs. 857 ± 217, *p* = 0.152) and in all regions in women (coronal, 2002 ± 319 vs. 1322 ± 204, *p* = 0.082, midway1 1684 ± 345 vs. 1379 ± 277, *p* = 0.417, midway2, 1330 ± 396 vs. 1372 ± 320, *p* = 0.915, apical, 1087 436 vs. 1354 338, *p* = 0.521).

## Discussion

**Fig. 9:**
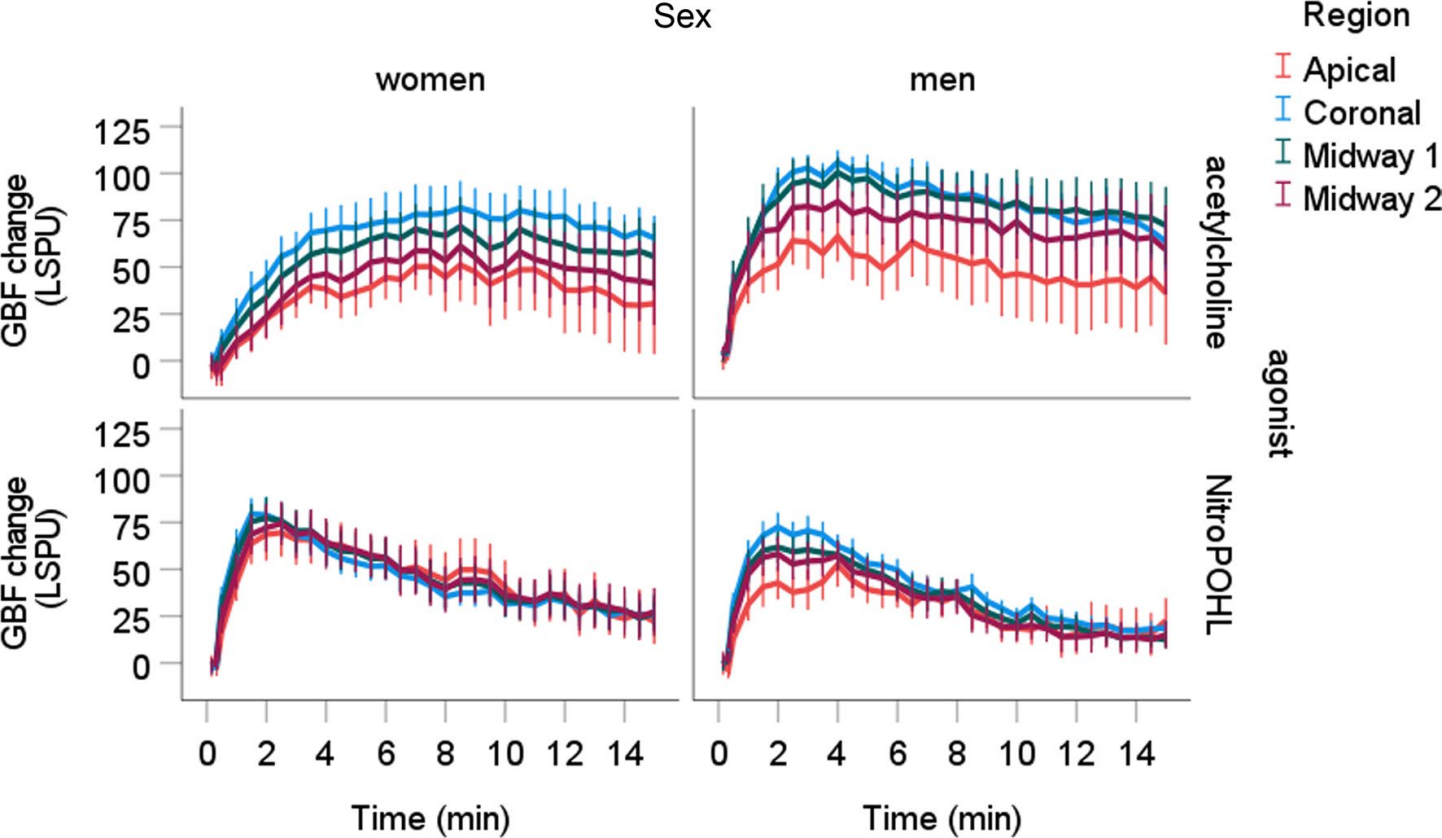
Blood flow changes from the baseline normalized to the saline site in different regions after acetylcholine or NitroPOHL administration in different regions

Both endothelium-dependent and independent local hyperemia elicited spreading vasodilation. The vestibule contains the upstream (supply) arteries of the attached and marginal gingiva as demonstrated by morphology [30, 31], by wounding study [32] and by occlusal test [5, 31]. Therefore, the observed spreading vasodilation was upstream in nature. Several mechanisms may be responsible for upstream vasodilation [1]. In vitro experiment on hamster cheek punch demonstrated that acetylcholine evoked conducted vasodilation was less than 50% of the local diameter changes within 1 mm [33, 34]. According to Poiseuille's law, this should result in even more significant attenuation in blood flow changes [35]. However, in this study, the apical (3 mm distance) AUC and the Dmax were 57% and 71% of the coronal one after acetylcholine administration, indicating that other mechanisms might also contribute to propagated vasodilation. Additionally, nitric oxide only causes localized vasodilation without conducted vasodilation [33, 34]. Therefore, the propagated gingival hyperemia could be explained by the FMD. The sheer stress of the apical endothelium wall might increase due to the higher velocity of the flow induced by the vasodilation at the marginal gingiva. The increased shear stress releases nitric oxide and prostacyclin from the endothelium resulting in vasodilation of the upstream arterioles in rat gracilis muscle [10]. Contrarily, cutaneous post-occlusive reactive hyperemia is mediated by a non-prostanoid [36] and non-nitric oxide mechanism [17]. Due to the differences between tissues and vessel sizes [37], it is difficult to suggest the main contributor to the FMD in the human gingiva. In cat gingiva, the reactive hyperemia could be suppressed by nitric oxide synthase inhibitors, especially the neural NO synthase selective antagonist, but not by anticholinergic drugs, β-blockers, or antihistaminic drugs [38]. In rat gingiva, the post-occlusive hyperemia was augmented after topical administration of acetylcholine or nitroglycerin [39, 40]. In dog gingiva, pretreatment with nitric oxide inhibitor suppressed the PORH [41]. Accordingly, the upstream vasodilation in the gingiva could be partially mediated by the nitric oxide; however, further pharmacology study is necessary to reveal the mechanism. The FMD requires 8–10 s to initiate dilation in upstream arterioles [42, 43]. In the gingiva, the apical vasodilation (3 mm away) followed the coronal one closely (Fig. 9), indicating a quick remote response. Therefore, the FMD could play a role in the apical vasodilation induced by both agonists. The larger arterioles are more sensitive to shear stress than the small ones [18]. In gingiva, the larger arterioles localized apically [30, 31, 44]; therefore, this might explain the lack of decay of the NitroPOHL effect.

In men, local (coronal and midway1) acetylcholine response was higher and faster than in women, but the difference was diminished at the remote sites (midway2 and apical). On the contrary, no sex difference was found after applying NitroPOHL. Our results indicate that endothelium-dependent gingival vasodilation is more pronounced in men than women. In men, the skin microcirculation in resting and after the PORH test was also higher [15]. The sex differences in gingival blood flow regulation might explain why males have improved wound healing after tooth extraction [45] and higher blood flow in the mucogingival flap [4]. However, the reason for the higher severity of periodontal disease in men than in women [8, 46] is more complicated to answer from our findings. In periodontitis, the endothelial-dependent vasodilation decreased in rat gingiva [39, 40]. It was investigated only in male rats; thus, the interaction between sex and periodontitis could not be revealed. Nevertheless, only the PORH was evaluated, but the baseline blood flow was not compared between control and periodontitis. In gingivitis, some studies [47–49] found higher resting GBF, which could lessen the vasodilator capacity. In the current and previous study [31], men had higher gingival baseline blood flow than women. We could only speculate that higher gingival blood flow and endothelial reactivity of males in healthy gingiva is also true for periodontitis. If so, the increased blood supply might exaggerate the destructive phase of periodontitis by increasing the inflammatory reaction. In rats, ligature-induced periodontitis increases the periodontal inducible nitric oxide synthase activity [50]. Patients with chronic periodontitis had higher nitric oxide synthase expression, including all isoform types, than patients with healthy gingiva [51]. Therefore, the pronounced endothelium-dependent gingival vasodilation in men and the high nitric oxide production in periodontitis might explain the higher severity of periodontal disease [8, 46].

The excess nitric oxide is detrimental to the periodontal tissues [52], contrarily to its protective effect in the systemic circulation [21, 27, 53]. The post-occlusive reactive hyperemia at the brachial artery is reduced in patients with advanced periodontitis [12]. Conversely, the PORH in the brachial artery was increased after intensive periodontal treatment compared to community-based periodontal care [54] and healthy controls [55]. More specifically, the acetylcholine-induced forearm blood flow is also decreased in periodontitis, but not after sodium nitroprusside application [56] or after nitroglycerin [54, 55], indicating endothelial dysfunction. Therefore, the periodontitis seems deteriorating endothelium-dependent vasodilation in the systemic circulation. Men develop cardiovascular disease 7–10 years earlier than women, but after menopause, the prevalence of cardiovascular disease increases in women [57, 58]. Therefore, the male sex should be considered an essential factor in assessing the risk of cardiovascular disease in periodontitis patients [59]. More aggressive periodontitis in males might worsen systemic effect.

The limitation of our study was that neither the sex hormone level nor the menstrual cycle was recorded. Estrogen facilitates agonists and shear stress-induced nitric oxide and prostacyclin production [60]. The estrogen enhances vasodilation by estrogen receptor-dependent and independent mechanisms in several ways [60]. An example is endothelial nitric oxide synthase and cyclooxygenase increase via receptor activation (genomic effect). In the cytoplasm (non-genomic effect), estrogen stimulates a series of intracellular pathways. It can also block the calcium channel on the vascular smooth muscle surface (estrogen receptor-independent manner). In isolated skeletal muscle arterioles, the estrogen attenuated myogenic response in female rats [61] by endothelium and nitric oxide dependent mechanism. The flow-induced vasodilation was higher in female isolated arteriole, which was estrogen-dependent and NO-mediated [62, 63]. Interestingly, if the NO pathway was eliminated, the female arterioles maintain the enhanced FMD via endothelium-derived hyperpolarizing factor [63]. In coronary circulation [64], estrogen enhanced microvascular sensitivity to acetylcholine. Similarly, males have lower acetylcholine-induced vascular relaxation in an isolated rat artery [65]. However, large artery and microcirculation have different responsiveness to the various regulation mechanisms (e.g., metabolic, myogenic, flow-induced, etc.) [18]. Overall, the high estrogen level in women should increase reactive hyperemia. However, in the skin, the PORH, measured in skin microcirculation, was similar in the low and high estrogen phases in women [16]. Nevertheless, women have lower testosterone and higher estrogen levels than men regardless of the menstrual phase [16, 66–68]. Therefore, the vascular effect of estrogen might not explain the higher endothelium-dependent vasodilation in the gingiva. Further study is necessary to reveal the mechanism behind the sex difference in endothelial-dependent gingival blood flow.

## Conclusion

The endothelium-independent vasodilation evoked by the nitric oxide donor could spread upstream (apically) in the gingiva without attenuation, contrary to acetylcholine-induced endothelium-dependent one. Nevertheless, endothelium-dependent vasodilation seems more pronounced in men than in women. The effect of sex on microcirculation regulation could explain the previously observed sex differences in oral flap blood flow and periodontitis.

## Data Availability

The datasets used and/or analysed during the current study are available from the corresponding author on reasonable request.

## Abbreviations

FMD: Flow-mediated vasodilation
LSPU: Laser Speckle Perfusion Unit
GBF: Gingival blood flow
GCF: Gingival crevicular fluid
Dmax: Absolute maximal blood flow change
AUC: The area under the blood flow curve
TTP: The time to peak
PORH: Post-occlusive reactive hyperemia test
BP: Blood pressure

## References

[1] Segal SS (2015). Integration and modulation of intercellular signaling underlying blood flow control. J Vasc Res.

[2] Ganti B, Molnar E, Fazekas R, Mikecs B, Lohinai Z, Miko S (2019). Evidence of spreading vasodilation in the human gingiva evoked by nitric oxide. J Periodontal Res.

[3] Vag J, Ganti B, Mikecs B, Szabo E, Molnar B, Lohinai Z (2020). Epinephrine penetrates through gingival sulcus unlike keratinized gingiva and evokes remote vasoconstriction in human. BMC Oral Health.

[4] Molnar E, Molnar B, Lohinai Z, Toth Z, Benyo Z, Hricisak L (2017). Evaluation of laser speckle contrast imaging for the assessment of oral mucosal blood flow following periodontal plastic surgery: an exploratory study. Biomed Res Int.

[5] Fazekas R, Molnar E, Lohinai Z, Dinya E, Toth Z, Windisch P (2018). Functional characterization of collaterals in the human gingiva by laser speckle contrast imaging. Microcirculation.

[6] Engeland CG, Sabzehei B, Marucha PT (2009). Sex hormones and mucosal wound healing. Brain Behav Immun.

[7] Peng X, Wang J, Lassance-Soares RM, Najafi AH, Sood S, Aghili N (2011). Gender differences affect blood flow recovery in a mouse model of hindlimb ischemia. Am J Physiol Heart Circ Physiol.

[8] Shiau HJ, Reynolds MA (2010). Sex differences in destructive periodontal disease: a systematic review. J Periodontol.

[9] Ibrahimi K, De Graaf Y, Draijer R, Jan Danser AH, Maassen VanDenBrink A, van den Meiracker AH (2018). Reproducibility and agreement of different non-invasive methods of endothelial function assessment. Microvasc Res.

[10] Koller A, Sun D, Huang A, Kaley G (1994). Corelease of nitric oxide and prostaglandins mediates flow-dependent dilation of rat gracilis muscle arterioles. Am J Physiol.

[11] Green DJ, Dawson EA, Groenewoud HM, Jones H, Thijssen DH (2014). Is flow-mediated dilation nitric oxide mediated?: A meta-analysis. Hypertension.

[12] Amar S, Gokce N, Morgan S, Loukideli M, Van Dyke TE, Vita JA (2003). Periodontal disease is associated with brachial artery endothelial dysfunction and systemic inflammation. Arterioscler Thromb Vasc Biol.

[13] Levenson J, Pessana F, Gariepy J, Armentano R, Simon A (2001). Gender differences in wall shear-mediated brachial artery vasoconstriction and vasodilation. J Am Coll Cardiol.

[14] Nishiyama SK, Wray DW, Richardson RS (2008). Sex and limb-specific ischemic reperfusion and vascular reactivity. Am J Physiol Heart Circ Physiol.

[15] Schlager O, Giurgea A, Hammer A, Charwat-Resl S, Margeta C, Mueller M (2014). Impact of age and gender on microvascular function. Eur J Clin Investig.

[16] Yvonne-Tee GB, Rasool AH, Halim AS, Wong AR, Rahman AR (2008). Method optimization on the use of postocclusive hyperemia model to assess microvascular function. Clin Hemorheol Microcirc.

[17] Wong BJ, Wilkins BW, Holowatz LA, Minson CT (1985). Nitric oxide synthase inhibition does not alter the reactive hyperemic response in the cutaneous circulation. J Appl Physiol.

[18] Davis MJ, Hill MA, Kuo L, Durán WN, Ley K (2008). Local regulation of microvascular perfusion. Microcirculation.

[19] Schonberger RB, Worden WS, Shahmohammadi K, Menn K, Silverman TJ, Stout RG (2006). Topical non-iontophoretic application of acetylcholine and nitroglycerin via a translucent patch: a new means for assessing microvascular reactivity. Yale J Biol Med.

[20] Ignarro LJ, Buga GM, Wood KS, Byrns RE, Chaudhuri G (1987). Endothelium-derived relaxing factor produced and released from artery and vein is nitric oxide. Proc Natl Acad Sci U S A.

[21] Divakaran S, Loscalzo J (2017). The role of nitroglycerin and other nitrogen oxides in cardiovascular therapeutics. J Am Coll Cardiol.

[22] Molnar E, Fazekas R, Lohinai Z, Toth Z, Vag J (2018). Assessment of the test-retest reliability of human gingival blood flow measurements by Laser Speckle Contrast Imaging in a healthy cohort. Microcirculation.

[23] Beer S, Feihl F, Ruiz J, Juhan-Vague I, Aillaud MF, Wetzel SG (2008). Comparison of skin microvascular reactivity with hemostatic markers of endothelial dysfunction and damage in type 2 diabetes. Vasc Health Risk Manag.

[24] Monostori P, Barath A, Fazekas I, Hodi E, Mate A, Farkas I (2010). Microvascular reactivity in lean, overweight, and obese hypertensive adolescents. Eur J Pediatr.

[25] Farkas K, Nemcsik J, Kolossvary E, Jarai Z, Nadory E, Farsang C (2005). Impairment of skin microvascular reactivity in hypertension and uraemia. Nephrol Dial Transplant.

[26] Farkas K, Kolossvary E, Jarai Z, Nemcsik J, Farsang C (2004). Non-invasive assessment of microvascular endothelial function by laser Doppler flowmetry in patients with essential hypertension. Atherosclerosis.

[27] Walther G, Obert P, Dutheil F, Chapier R, Lesourd B, Naughton G (2015). Metabolic syndrome individuals with and without type 2 diabetes mellitus present generalized vascular dysfunction: cross-sectional study. Arterioscler Thromb Vasc Biol.

[28] Vickers AJ (2001). The use of percentage change from baseline as an outcome in a controlled trial is statistically inefficient: a simulation study. BMC Med Res Methodol.

[29] Zhang L, Han K. How to analyze change from baseline: absolute or percentage change?. D-level essay in statistics 2009.

[30] Nobuto T, Yanagihara K, Teranishi Y, Minamibayashi S, Imai H, Yamaoka A (1989). Periosteal microvasculature in the dog alveolar process. J Periodontol.

[31] Mikecs B, Vag J, Gerber G, Molnar B, Feigl G, Shahbazi A (2021). Revisiting the vascularity of the keratinized gingiva in the maxillary esthetic zone. BMC Oral Health.

[32] Fazekas R, Molnar B, Kohidai L, Lang O, Molnar E, Ganti B (2019). Blood flow kinetics of a xenogeneic collagen matrix following a vestibuloplasty procedure in the human gingiva-an explorative study. Oral Dis.

[33] Budel S, Bartlett IS, Segal SS (2003). Homocellular conduction along endothelium and smooth muscle of arterioles in hamster cheek pouch: unmasking an NO wave. Circ Res.

[34] Delashaw JB, Duling BR (1991). Heterogeneity in conducted arteriolar vasomotor response is agonist dependent. Am J Physiol.

[35] Secomb TW (2008). Theoretical models for regulation of blood flow. Microcirculation.

[36] Hellmann M, Gaillard-Bigot F, Roustit M, Cracowski JL (2015). Prostanoids are not involved in postocclusive reactive hyperaemia in human skin. Fundam Clin Pharmacol.

[37] Davis CM, Siler DA, Alkayed NJ (2011). Endothelium-derived hyperpolarizing factor in the brain: influence of sex, vessel size and disease state. Womens Health (Lond).

[38] Shimada S, Todoki K, Omori Y, Toyama T, Matsuo M, Wada-Takahashi S (2015). Contribution of nitrergic nerve in canine gingival reactive hyperemia. J Clin Biochem Nutr.

[39] Funaki S, Tokutomi F, Wada-Takahashi S, Yoshino F, Yoshida A, Maehata Y (2016). Porphyromonas gingivalis infection modifies oral microcirculation and aortic vascular function in the stroke-prone spontaneously hypertensive rat (SHRSP). Microb Pathog.

[40] Sugiyama S, Takahashi SS, Tokutomi FA, Yoshida A, Kobayashi K, Yoshino F (2012). Gingival vascular functions are altered in type 2 diabetes mellitus model and/or periodontitis model. J Clin Biochem Nutr.

[41] Omori Y, Takahashi SS, Todoki K (2002). Role of nitric oxide in post-ischemic gingival hyperemia in anesthetized dogs. Redox Rep.

[42] Koller A, Kaley G (1990). Endothelium regulates skeletal muscle microcirculation by a blood flow velocity-sensing mechanism. Am J Physiol.

[43] Smiesko V, Johnson P (1993). The arterial lumen is controlled by flow-related shear stress. Physiology.

[44] Nuki K, Hock J (1974). The organisation of the gingival vasculature. J Periodontal Res.

[45] Adeyemo WL, Ladeinde AL, Ogunlewe MO (2006). Clinical evaluation of post-extraction site wound healing. J Contemp Dent Pract.

[46] Stanescu I, Bulboaca AE, Micu IC, Bolboaca SD, Festila DG, Bulboaca AC (2020). Gender differences in the levels of periodontal destruction, behavioral risk factors and systemic oxidative stress in ischemic stroke patients: a cohort pilot study. J Clin Med.

[47] Gleissner C, Kempski O, Peylo S, Glatzel JH, Willershausen B (2006). Local gingival blood flow at healthy and inflamed sites measured by laser Doppler flowmetry. J Periodontol.

[48] Hock JM, Kim S (1987). Blood flow in healed and inflamed periodontal tissues of dogs. J Periodontal Res.

[49] Kaplan ML, Jeffcoat MK, Goldhaber P (1982). Blood flow in gingiva and alveolar bone in beagles with periodontal disease. J Periodontal Res.

[50] Lohinai Z, Benedek P, Feher E, Gyorfi A, Rosivall L, Fazekas A (1998). Protective effects of mercaptoethylguanidine, a selective inhibitor of inducible nitric oxide synthase, in ligature-induced periodontitis in the rat. Br J Pharmacol.

[51] Artese L, Piattelli A, de Gouveia Cardoso LA, Ferrari DS, Onuma T, Piccirilli M (2010). Immunoexpression of angiogenesis, nitric oxide synthase, and proliferation markers in gingival samples of patients with aggressive and chronic periodontitis. J Periodontol.

[52] Lohinai Z, Szabo C (1998). Role of nitric oxide in physiology and patophysiology of periodontal tissues. Med Sci Monit.

[53] Morishita T, Tsutsui M, Shimokawa H, Horiuchi M, Tanimoto A, Suda O (2002). Vasculoprotective roles of neuronal nitric oxide synthase. FASEB J.

[54] Tonetti MS, D'Aiuto F, Nibali L, Donald A, Storry C, Parkar M (2007). Treatment of periodontitis and endothelial function. N Engl J Med.

[55] Blum A, Kryuger K, Mashiach Eizenberg M, Tatour S, Vigder F, Laster Z (2007). Periodontal care may improve endothelial function. Eur J Intern Med.

[56] Higashi Y, Goto C, Jitsuiki D, Umemura T, Nishioka K, Hidaka T (2008). Periodontal infection is associated with endothelial dysfunction in healthy subjects and hypertensive patients. Hypertension.

[57] Wakabayashi I (2017). Gender differences in cardiovascular risk factors in patients with coronary artery disease and those with type 2 diabetes. J Thorac Dis.

[58] Maas AH, Appelman YE (2010). Gender differences in coronary heart disease. Neth Heart J.

[59] Roeters van Lennep JE, Westerveld HT, Erkelens DW, van der Wall EE (2002). Risk factors for coronary heart disease: implications of gender. Cardiovasc Res.

[60] Huang A, Kaley G (2004). Gender-specific regulation of cardiovascular function: estrogen as key player. Microcirculation.

[61] Huang A, Sun D, Koller A, Kaley G (1997). Gender difference in myogenic tone of rat arterioles is due to estrogen-induced, enhanced release of NO. Am J Physiol.

[62] Huang A, Sun D, Koller A, Kaley G (1998). Gender difference in flow-induced dilation and regulation of shear stress: role of estrogen and nitric oxide. Am J Physiol.

[63] Huang A, Sun D, Carroll MA, Jiang H, Smith CJ, Connetta JA (2001). EDHF mediates flow-induced dilation in skeletal muscle arterioles of female eNOS-KO mice. Am J Physiol Heart Circ Physiol.

[64] Thompson LP, Pinkas G, Weiner CP (2000). Chronic 17beta-estradiol replacement increases nitric oxide-mediated vasodilation of guinea pig coronary microcirculation. Circulation.

[65] Sipos M, Gerszi D, Dalloul H, Banyai B, Sziva RE, Kollarics R (2021). Vitamin D deficiency and gender alter vasoconstrictor and vasodilator reactivity in rat carotid artery. Int J Mol Sci.

[66] Södergard R, Bäckström T, Shanbhag V, Carstensen H (1982). Calculation of free and bound fractions of testosterone and estradiol-17β to human plasma proteins at body temperature. J Steroid Biochem.

[67] Andersson B, Märin P, Lissner L, Vermeulen A, Björntorp P (1994). Testosterone concentrations in women and men with NIDDM. Diabetes Care.

[68] Clapauch R, Mecenas AS, Maranhao PA, Bouskela E (2009). Microcirculatory function in postmenopausal women: role of aging, hormonal exposure and metabolic syndrome. Microvasc Res.

